# Ovarian hormones and high‐fat diet duration distinctively modulate hypothalamic chemokine profile

**DOI:** 10.1111/jne.70215

**Published:** 2026-06-25

**Authors:** Alexia Guimaraes Batista Augusto, Giovanna Ariozi dos Santos, Igor Vinicius Sousa Cavalheiro, Vinicius Stefanini Mantovan, Larissa Silva Bergantini, Nicolly Porto Marin, Ester dos Santos Alves, Licio Augusto Velloso, Natalia Ferreira Mendes

**Affiliations:** ^1^ Laboratory of Cell Signaling, Obesity and Comorbidities Research Center University of Campinas Campinas Brazil; ^2^ School of Medical Sciences, Department of Translational Medicine—Section of Pharmacology University of Campinas Campinas Brazil; ^3^ School of Medical Sciences, Department of Pathophysiology University of Campinas Campinas Brazil; ^4^ National Institute of Science and Technology on Neuroimmunomodulation Rio de Janeiro Brazil

**Keywords:** chemokines, energy balance, estrogen, hypothalamus, sex dimorphism

## Abstract

Excessive intake of saturated fats triggers inflammation in the hypothalamus, a key regulator of energy balance. In the chronic phase of this inflammatory response, bone marrow‐derived and lymphoid cells are chemoattracted to this region, partially mitigating high‐fat diet (HFD)‐induced metabolic impairments. In rodents, the onset and magnitude of this inflammation differ between males and females, reflecting sex‐specific patterns of metabolic regulation. However, how the hypothalamic chemokine profile evolves during HFD‐induced inflammation, and whether it is influenced by biological sex, remains unclear. Here, male and female C57BL/6J mice were fed a HFD for 1, 3, 14, or 28 days. To isolate the role of ovarian hormones in modulating hypothalamic chemokine profile, we also analyzed ovariectomized (OVX) females with or without estrogen replacement. Quantitative polymerase chain reaction‐based expression analysis revealed that most chemokines and their receptors were transiently modulated in the hypothalamus during the course of the HFD exposure, showing reduced levels in the acute phase and normalization during the chronic phase. Despite the modest sex‐dependent effects observed, messenger RNA expression of the chemokine receptor C–X–C motif chemokine receptor 3 (CXCR3) was significantly higher in females than in males after 14 and 28 days of HFD, suggesting faster recruitment of CXCR3^+^ immune cells that may contribute to female protection against metabolic dysfunction. Females lacking ovarian hormone production displayed increased hypothalamic expression of *Cxcr3* and *Ccl2*. Only *Cxcr3* expression was partially normalized by estradiol treatment, suggesting that non‐estrogenic ovarian factors may play a role in modulating specific chemokine signaling pathways. Together, our findings show that hypothalamic chemokine signaling is dynamically and transiently regulated throughout the phases of HFD‐induced inflammation, with *Cxcr3* modulation by HFD and ovarian hormones contributing to sex‐specific resilience against metabolic inflammation.

## 1 INTRODUCTION

As a central regulator of energy homeostasis, the hypothalamus integrates peripheral and central cues, including hormonal and nutritional signals, through coordinated activity of anorexigenic and orexigenic neurons.^1^, ^2^ A diet rich in saturated fatty acids (SFAs) rapidly disrupts this hypothalamic neuronal network involved with control of food intake and energy expenditure, contributing to fat mass accumulation.^3^, ^4^ High‐fat diet (HFD)‐induced hypothalamic dysfunction arises from a transient inflammatory response that evolves in distinct temporal phases.^5^, ^6^ In the acute phase, glial cells release high levels of pro‐inflammatory cytokines and undergo morphological changes, a process known as gliosis.^7^, ^8^ Perivascular macrophages contribute to the inflammatory milieu by producing inducible nitric oxide synthase (iNOS) and vascular endothelial growth factor (VEGF), which promote angiogenesis and increase vascular permeability.^9^, ^10^, ^11^ Following this initial phase, there is an adaptive transition, during which compensatory mechanisms attempt to restore homeostasis.^5^ Chronic phase is marked by the establishment of central resistance to leptin and insulin, further disrupting energy homeostasis and exacerbating metabolic dysregulation.^6^, ^12^, ^13^, ^14^

Emerging evidence highlights that the chronic stage of HFD‐induced hypothalamic inflammation is accompanied by the directed migration of peripheral immune cells, such as myeloid cells originating from bone marrow progenitors, and Forkhead box P3 (Foxp3^+^) regulatory T cells (Tregs) derived from the thymus.^15^, ^16^, ^17^, ^18^ Four weeks of HFD exposure triggers the infiltration of bone marrow‐derived cluster of differentiation 169 (CD169^+^) immune cells into the mediobasal hypothalamus via the fenestrated vasculature of the median eminence (ME).^15^ Upon entry, these cells migrate toward the arcuate nucleus (ARC) and undergo morphological transformation, adopting a microglia‐like phenotype; however, their precise role in HFD‐induced hypothalamic inflammation remains unclear. Recent data indicate that Foxp3^+^ Tregs C–X–C motif chemokine receptor 3 (CXCR3^hi^), with lymphoid origin, exert a protective effect in this inflammatory context, as their depletion exacerbates HFD‐induced hypothalamic immune activation.^16^ Similarly, our group has recently shown that CXCR3^+^ myeloid cells help mitigate HFD‐induced metabolic impairments.^17^ In this study, we have also found that recruited hypothalamic C–C chemokine receptor type 2 (CCR2^+^) cells exhibit pronounced sex‐specific messenger RNA (mRNA) expression, including differential expression of genes involved in chemokine signaling and immune response pathways, suggesting that biological sex modulates their contribution to HFD‐induced hypothalamic inflammation.

Sex differences in the architecture and function of the central melanocortin system have been increasingly recognized and may underlie the distinct metabolic impairments observed between males and females in obesity and other metabolic disorders.^19^ However, how biological sex shapes the hypothalamic chemokine profile during the progression of HFD‐induced inflammatory response remains unexplored. We hypothesized that hypothalamic chemokine and chemokine receptor expression is dynamically and differentially regulated throughout the phases of HFD‐induced inflammation in a sex‐dependent manner. To test this, we analyzed the transcripts of the hypothalamus of male and female mice subjected to acute and chronic HFD, as well as ovariectomized females fed a standard diet (SD) with or without estradiol (EST) replacement. We demonstrated that most chemokine pathways show biphasic regulation during HFD exposure, independent of sex, with C–C motif chemokine ligand 2 (also known as MCP‐1) (CCL2)/CCR2 exhibiting particularly pronounced dynamics. In contrast, *Cxcr3* is sex‐specifically regulated, with higher female expression and sensitivity to dietary fat and ovarian hormones, revealing a critical role for ovarian hormones in hypothalamic immune homeostasis.

## 2 MATERIALS AND METHODS

### 2.1 Animal care and diets

Six‐week‐old male and female C57BL/6J mice were obtained from CEMIB (Centro Multidisciplinar para Investigação Biológica—University of Campinas, Brazil). All animal care and experimental procedures were conducted in accordance with the guidelines of the Brazilian College for Animal Experimentation and were approved by the Institutional Animal Care and Use Committee (CEUA 6393‐1/2024 and 6610‐1/2025).

For experiment with a dietary challenge, mice were socially housed (*N* = 2–3 per cage) in a controlled environment (22 ± 2°C, 12‐h light/dark cycle) with ad libitum access to food and filtered water. After 1 week of acclimatization, mice were randomly assigned to experimental groups receiving a HFD for 1, 3, 14, or 28 days (groups 1d HFD, 3d HFD, 14d HFD, and 28d HFD, respectively). The HFD provided 20.1% of energy from protein, 34.7% from carbohydrate, and 45.2% from fat (4.70 kcal/g; D12451, ResearchDiets, USA). Control group was fed exclusively with a SD containing 12.6% of energy from protein, 77.7% from carbohydrate, and 9.58% from fat (3.76 kcal/g; AIN93M, Pragsoluções, Brazil).

**FIGURE 1:**
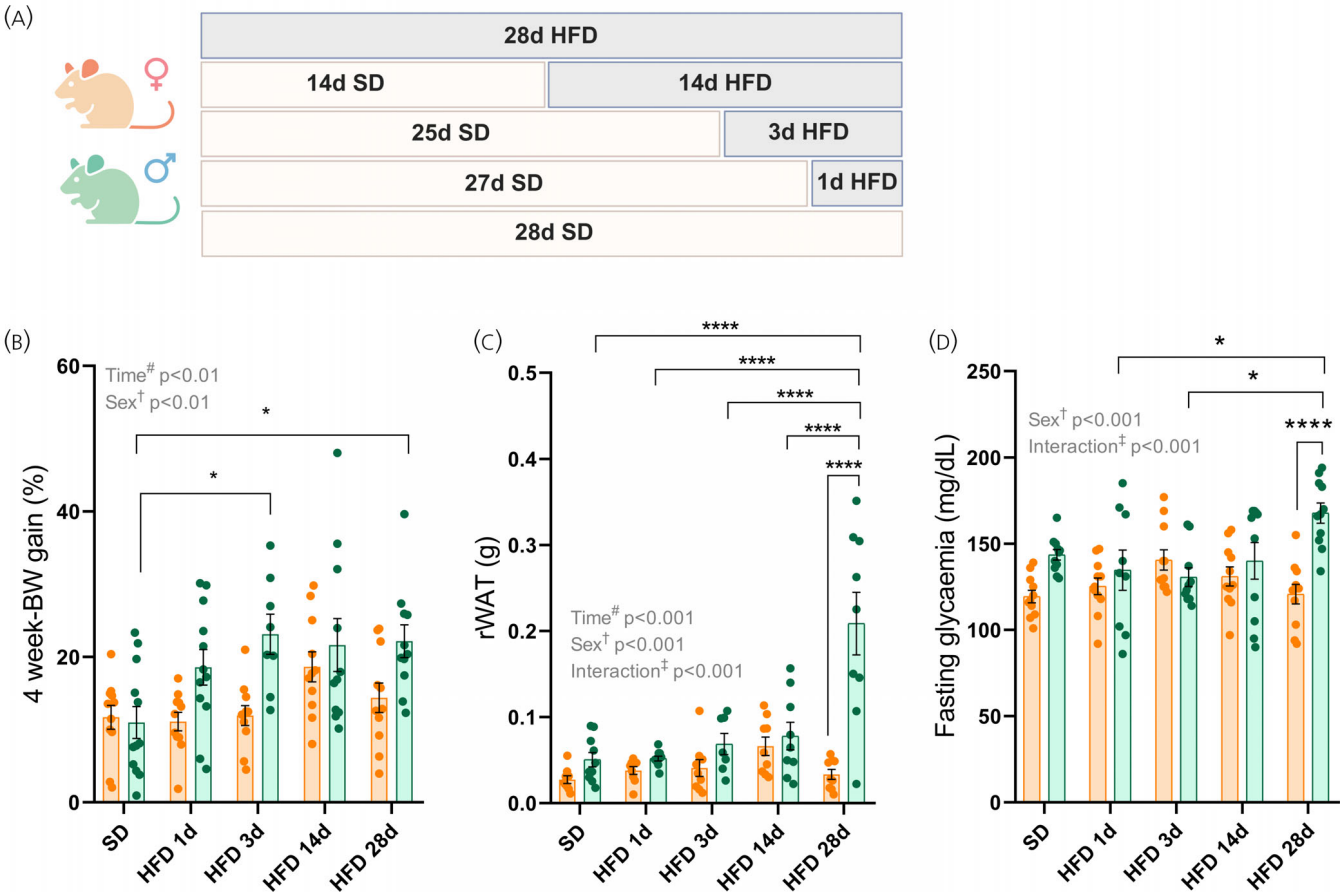
High‐fat diet induces sex‐specific metabolic alterations. (A) Schematic representation of the experimental protocol of the high‐fat diet (HFD) challenge. (B) Percentage of body weight gain during the experimental protocol. (C) Weight of white adipose tissue (retroperitoneal depot) at the end of the experiment. (D) Six‐hour fasting blood glucose levels. Data are expressed as mean ± standard error of the mean of 10–12 mice per group. Statistical analyses were performed using two‐way ANOVA followed by Sidak's post hoc test. *p < .05, ****p < .0001 compared with the indicated groups. BW, body weight; rWAT, retroperitoneal white adipose tissue; SD, standard diet. dagger: *p* < .05 when the effect of sex is observed by two‐way ANOVA analysis. double dagger: *p* < .05 when the effect of interaction of sex and time (HFD duration) is observed by two‐way ANOVA analysis.

The HFD challenge was conducted during the final days prior to euthanasia, according to the experimental timeline, ensuring that all mice were sacrificed at the same age (Figure 1A). Body weight (BW) was measured at baseline and at the end of the dietary intervention, on the day of euthanasia. On that day, mice were fasted from 8 a.m. to 2 p.m., and tissue collection was performed at 2 p.m.

### 2.2 Ovariectomy procedure and estradiol replacement

To analyze the role of ovarian hormones in hypothalamic chemokine profile, we conducted a bilateral ovariectomy procedure. The surgery was conducted as described by Mendes et al.^17^ The experimental timeline is shown in Figure 5A. Eight‐week‐old C57BL/6J female mice were anesthetized with a ketamine/xylazine solution (100 and 10 mg/kg, respectively). The ventral abdominal area was shaved and sterilized with iodine solution. A small midline incision was made to expose and bilaterally excise the ovaries (OVX group). Half of the ovariectomized mice received EST replacement via subcutaneously implanted 17β‐estradiol pellets (0.05 mg/pellet, 60‐day sustained release; Innovative Research of America, Inc., USA) beneath the dorsal neck region (OVX + EST group). Sham‐operated mice, which underwent the same procedure without ovary removal, were used as controls (Sham group). For analgesia, tramadol hydrochloride (5 mg/kg) was intraperitoneally administered immediately after surgery, and again after 24 and 36 h. Mice were individually housed and monitored during a 7‐day recovery period. From Day 7 to Day 35, we measured BW weekly. On Day 25, mice were fasted for 6 h, and fasting glycemia was measured prior to euthanasia.

### 2.3 Tissue sampling

Prior to tissue collection, mice were deeply anesthetized by intraperitoneal injection of a ketamine/xylazine solution (300 and 30 mg/kg, respectively). The hypothalamus was immediately harvested and frozen in liquid nitrogen. Retroperitoneal white adipose tissue (rWAT) was dissected and weighed. To confirm the efficacy of bilateral ovariectomy and EST replacement, the whole uterus from three mice of each group was collected, weighed, photographed for macroscopic comparison, and processed for histological analysis.

### 2.4 Uterus morphology

Uterine horns from groups Sham, OVX, and OVX + EST (*N* = 3/group) were fixed in 4% paraformaldehyde, paraffin‐embedded, and sectioned at 5 μm. Sections were stained with hematoxylin and eosin (H&E) following standard procedures. Images were acquired using an inverted brightfield microscope (Leica DMI 4000 B) at 10× and 40× magnifications at the National Institute of Photonics Applied to Cell Biology (INFABIC) at the University of Campinas.

### 2.5 Quantitative reverse transcription‐polymerase chain reaction

Hypothalami were homogenized in 500 μL of TRIzol Reagent (Invitrogen) and incubated for 5 min at reverse transcription (RT). After adding 100 μL of chloroform (Merck Millipore) and centrifuging at 12,000 rpm for 15 min at 4°C, the aqueous phase was collected and RNA precipitated with 250 μL of isopropanol. Following a 5‐min incubation at RT, samples were centrifuged (12,000 rpm, 4°C) to form RNA pellets, which were washed sequentially with 75% and 100% ethanol (8400 rpm, 10 min, 4°C), air‐dried, and resuspended in 20 μL RNase/DNase‐free water. For reverse transcription, 2 μg of RNA was converted to complementary DNA (cDNA) using the High‐Capacity cDNA Reverse Transcription Kit (Life Technologies) following the manufacturer's protocol. RNA quantity and purity were measured by absorbance at 260/280 nm.

Real‐time polymerase chain reaction (PCR) was performed using the TaqMan system (Applied Biosystems). cDNA products were diluted 1:10, and a pooled mix was used to generate a standard curve. On a 96‐well plate, each well received 3.5 μL of TaqMan mix (PCR Biosystems, qPCRBIO Probe Mix), 0.25 μL of DNase/RNase‐free water, 0.25 μL of primers, and 4 μL of diluted cDNA. Gene expression was measured using QuantStudio 6 (Applied Biosystems).

Cycle threshold (Ct) values were obtained using QuantStudio software (Applied Biosystems). Relative mRNA expression levels of target genes were normalized to the housekeeping gene Gapdh (Mm99999915_g1) using the ΔCt method. For comparisons between experimental groups, the ΔΔCt method was applied, and fold changes were calculated as 2^−ΔΔCt^. Gene expression levels are presented as log_2_ fold change relative to the mean of the respective control groups for data visualization and statistical analysis. Primer sequences were obtained from Thermo Fisher Scientific. The list of primers and their assay IDs are shown in Table S1.

### 2.6 Statistical analyses

Statistical analyses were conducted using GraphPad Prism version 8.4.2 https://www.graphpad.com/. For the diet challenge experiment, biometric parameters were compared using a two‐way analysis of variance (ANO with HFD duration and sex as factors, followed by Sidak's post hoc tests. Quantitative polymerase chain reaction (qPCR) data transformed to log_2_ fold change values were analyzed using two‐way ANOVA with HFD duration and sex as factors, followed by Sidak's post hoc tests. For the ovariectomy experiment, biometric parameters and qPCR were compared using a one‐way ANOVA, followed by Tukey's post hoc tests. Biometric data are presented as mean ± standard error of the mean, while qPCR data are shown as violin plots highlighting the median and quartiles. Significance is indicated as *p < .05, **p < .01, ***p < .001, and ****p < .0001.

## 3 RESULTS

### 3.1 High‐fat diet exposure triggers dynamic, sex‐dependent metabolic alterations and transient hypothalamic inflammation

HFD‐fed males showed greater BW gain and increased rWAT mass than HFD‐fed females, particularly after 28 days of HFD (*p* < .01; Figure 1B,C). Fasting glycemia was elevated in males of HFD 28d group (*p* < .001; Figure 1D). Despite these metabolic differences, caloric intake did not differ between HFD‐fed groups or sexes (data not shown).

**FIGURE 2:**
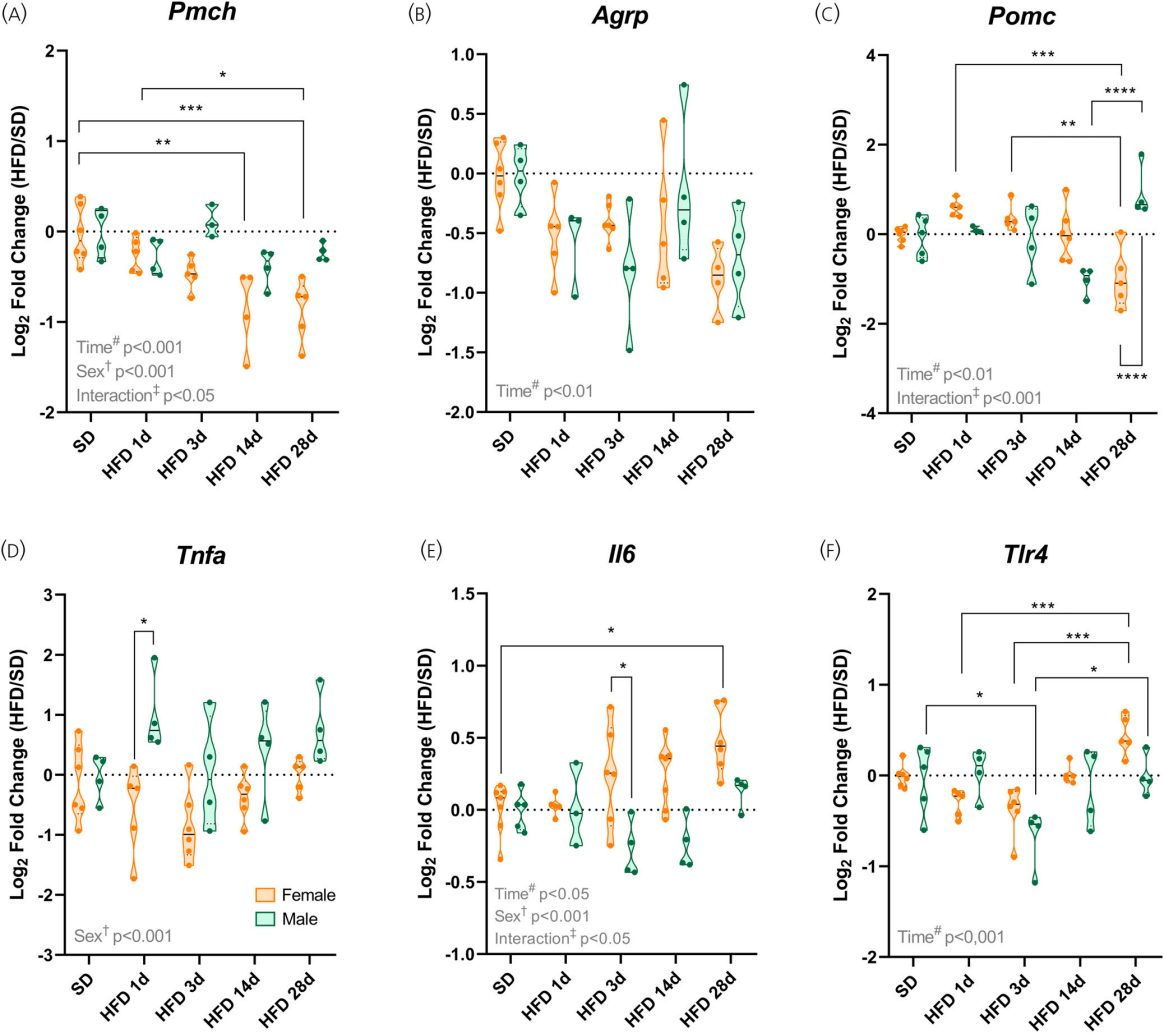
High‐fat intake induces hypothalamic inflammation and alters the neuropeptide profile in a sex‐ and time‐dependent manner. (A–C) Hypothalamic mRNA levels of neuropeptides. (D–F) Hypothalamic mRNA levels of inflammatory genes. Data are shown as violin plots with median and quartiles for four to six mice per group. qPCR data (log_2_ fold change) were analyzed using Two‐way ANOVA with sex and high‐fat diet (HFD) duration as factors, followed by Sidak's post hoc tests. *p < .05, **p < .01, ***p < .001, ****p < .0001 compared with the indicated groups. SD, standard diet.

Analysis of hypothalamic neuropeptide expression revealed that pro‐melanin concentrating hormone (*Pmch*) was consistently reduced in females compared to males throughout the HFD challenge, showing significant effects of sex, time, and their interaction (*p* < .05; Figure 2A). Agouti‐related peptide (*Agrp*) expression was slightly decreased in most HFD‐fed groups, primarily in a time‐dependent manner (*p* < .01), with no significant sex effects (Figure 2B). In females, pro‐opiomelanocortin (*Pomc*) expression declined only after 28 days of HFD (*p* < .01; Figure 2C), while males exhibited a non‐significant decrease at 14 days followed by a marked rebound at 28 days (*p* < .0001; Figure 2C).

The pro‐inflammatory marker tumor necrosis factor‐alpha (*Tnfa*) was higher in the hypothalamus of males at all time points, with the largest difference at 1 day of HFD (*p* < .05; Figure 2D). Conversely, interleukin‐6 (*Il6*) increased progressively in females, with significant effects of sex, diet duration, and their interaction for *Il6* (*p* < .05; Figure 2E). Toll‐like receptor 4 (*Tlr4*) expression was influenced by HFD duration, displaying a distinct biphasic pattern: reduced in the acute phase and increased in the chronic phase (*p* < .05; Figure 2F). Together, these results show that HFD‐fed male mice exhibit higher hypothalamic *Tnfa* expression than females at the early stage of diet exposure. These findings indicate a more pronounced early hypothalamic inflammatory response in males. Additionally, the temporal analysis reveals that the hypothalamic inflammatory response to HFD is dynamic and differs between sexes.

### 3.2 HFD exposure dynamically modulates hypothalamic chemokine pathways in a time‐dependent manner

**FIGURE 3:**
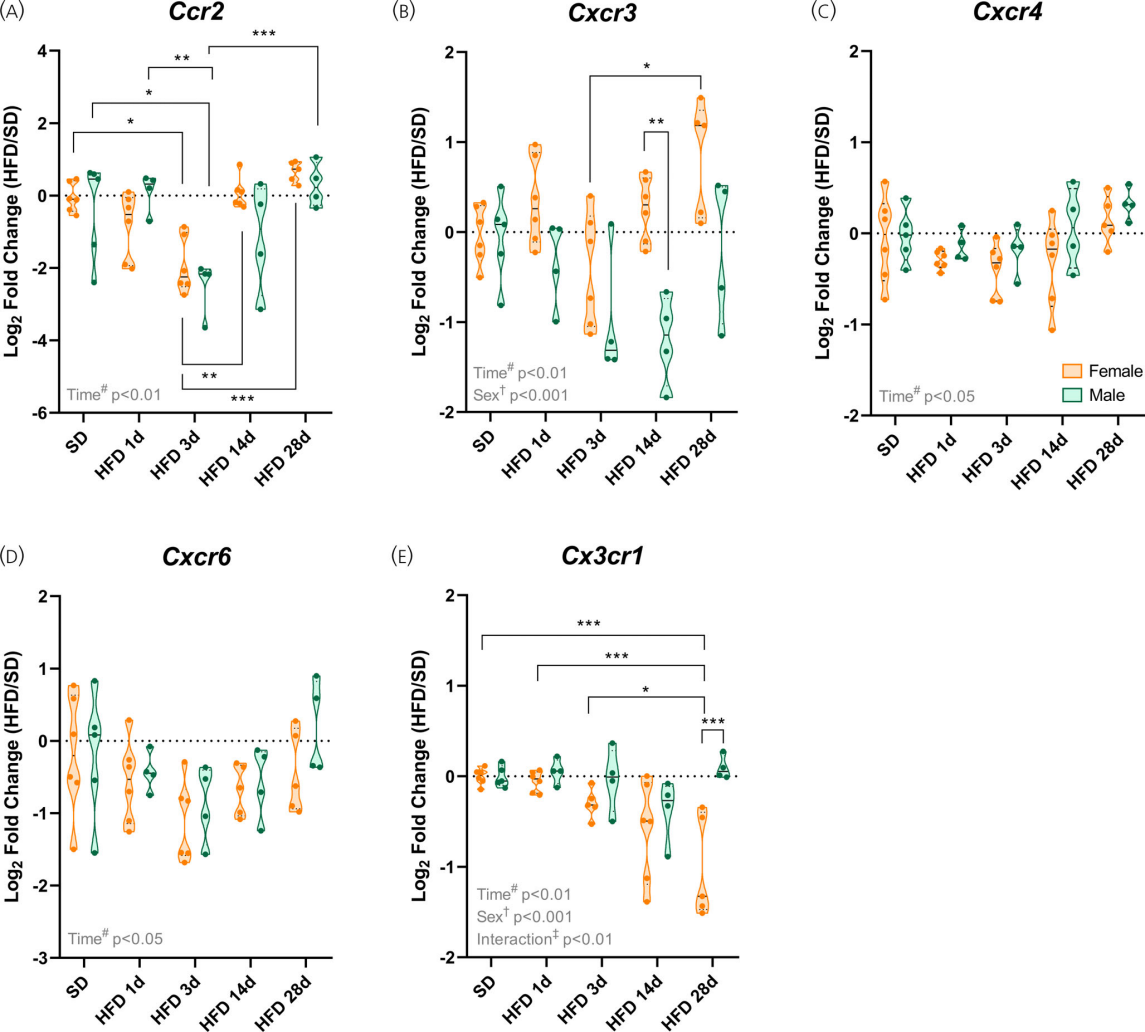
Chemokine receptors *Ccr2*, *Cxcr3*, and *Cx3cr1* are modulated by high‐fat diet (HFD) in females. (A–E) Hypothalamic mRNA levels of chemokine receptors. Data are shown as violin plots with median and quartiles for four to six mice per group. qPCR data (log_2_ fold change) were analyzed using two‐way ANOVA with sex and high‐fat diet (HFD) duration as factors, followed by Sidak's post hoc tests. *p < .05, **p < .01, ***p < .001 compared with the indicated groups. SD, standard diet.

**FIGURE 4:**
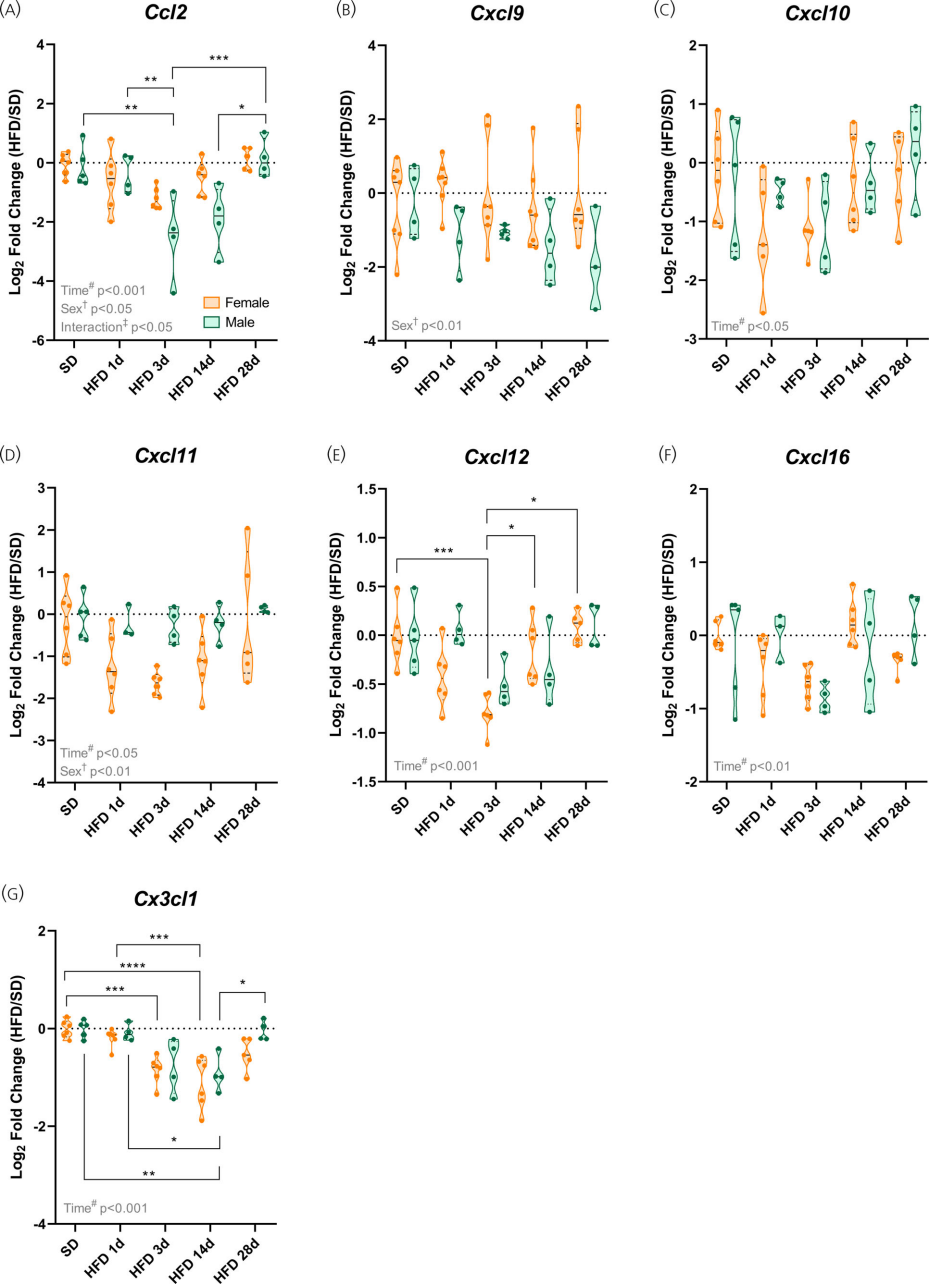
High‐fat diet induces sex‐specific modulation of the chemokines *Ccl2* and *Cx3cl1*. (A–G) Hypothalamic mRNA levels of various chemokines. Data are shown as violin plots with median and quartiles for four to six mice per group. qPCR data (log_2_ fold change) were analyzed using two‐way ANOVA with sex and high‐fat diet (HFD) duration as factors, followed by Sidak's post hoc tests. *p < .05, **p < .01, ***p < .001, ****p < .0001 compared with the indicated groups. SD, standard diet.

To better understand how hypothalamic chemokine profile changes in the distinct phases of HFD‐induced inflammation, we also evaluated the gene expression of some chemokines and their receptors. For both sexes, *Ccr*2, a chemokine receptor primarily expressed on key immune cells involved in inflammation and immune surveillance, and its ligand *Ccl2*, showed reduced expression during the acute phase of HFD challenge, followed by recovery in the chronic phase (*p* < .05; Figures 3A and 4A).

Similarly, *Cxcr3*, a chemokine receptor predominantly expressed on activated T cells, and its ligands (C–X–C motif chemokine ligand 9 [*Cxcl9*], C–X–C motif chemokine ligand 10 [*Cxcl10*], and C‐X‐C motif chemokine ligand 11 [*Cxcl11*]) displayed a transient decrease after 3 days of HFD exposure, followed by later upregulation, with females showing an earlier rise and higher expression of *Cxcr3* at 14 and 28 days of HFD (*p* < .05; Figures 3B and 4B–D).

The C–X–C motif chemokine receptor 4 (*Cxcr4*), a chemokine receptor expressed not only in infiltrating immune cells, but also in brain resident cells, and its ligand C–X–C motif chemokine ligand 12 [*Cxcl12*] (also known as stromal cell‐derived factor 1 alpha [SDF1‐α]) mirrored this pattern: *Cxcl12* decreased after 3 days of HFD in females and increased with chronic HFD (*p* < .001; Figure 4E), while *Cxcr4* showed time‐dependent changes without post hoc significance (*p* < .05; Figure 3C). The C–X–C motif chemokine receptor 6 (*Cxcr6*), a chemokine receptor involved in immune cell trafficking, and its ligand C–X–C motif chemokine ligand 16 (*Cxcl16*) were similarly affected by HFD duration, regardless of sex (*p* < .05; Figures 3D and 4F).

In contrast, the receptor *Cx3cr1*, a classical microglia marker, and *Cx3cl1* (fractalkine), which is its ligand, being primarily produced by neurons, displayed sex‐specific regulation. Females showed a progressive decline in *Cx3cr1* expression over time, whereas males maintained higher levels at 28 days of HFD (*p* < .001; Figure 3E). Corresponding changes in the ligand *Cx3cl1* reflected these dynamics, with early decreases in females and late increases in males (*p* < .001; Figure 4G).

Overall, these findings indicate that HFD induces transient alterations in hypothalamic chemokine signaling, such as in CCL2‐CCR2 and C–X3–C motif chemokine ligand 1‐C–X3–C motif chemokine receptor 1 (CX3CL1‐CX3CR1) pathways, following time‐dependent dynamics in key pathways involved in immune cell recruitment and neuron–microglia communication. Sex effects were not significant, except for *Cxcr3* and *Cx3cr1*, whose expression was, respectively, higher and lower in females during the chronic phase of HFD exposure.

### 3.3 Ovarian hormones absence triggers metabolic impairments and hypothalamic inflammation

**FIGURE 5:**
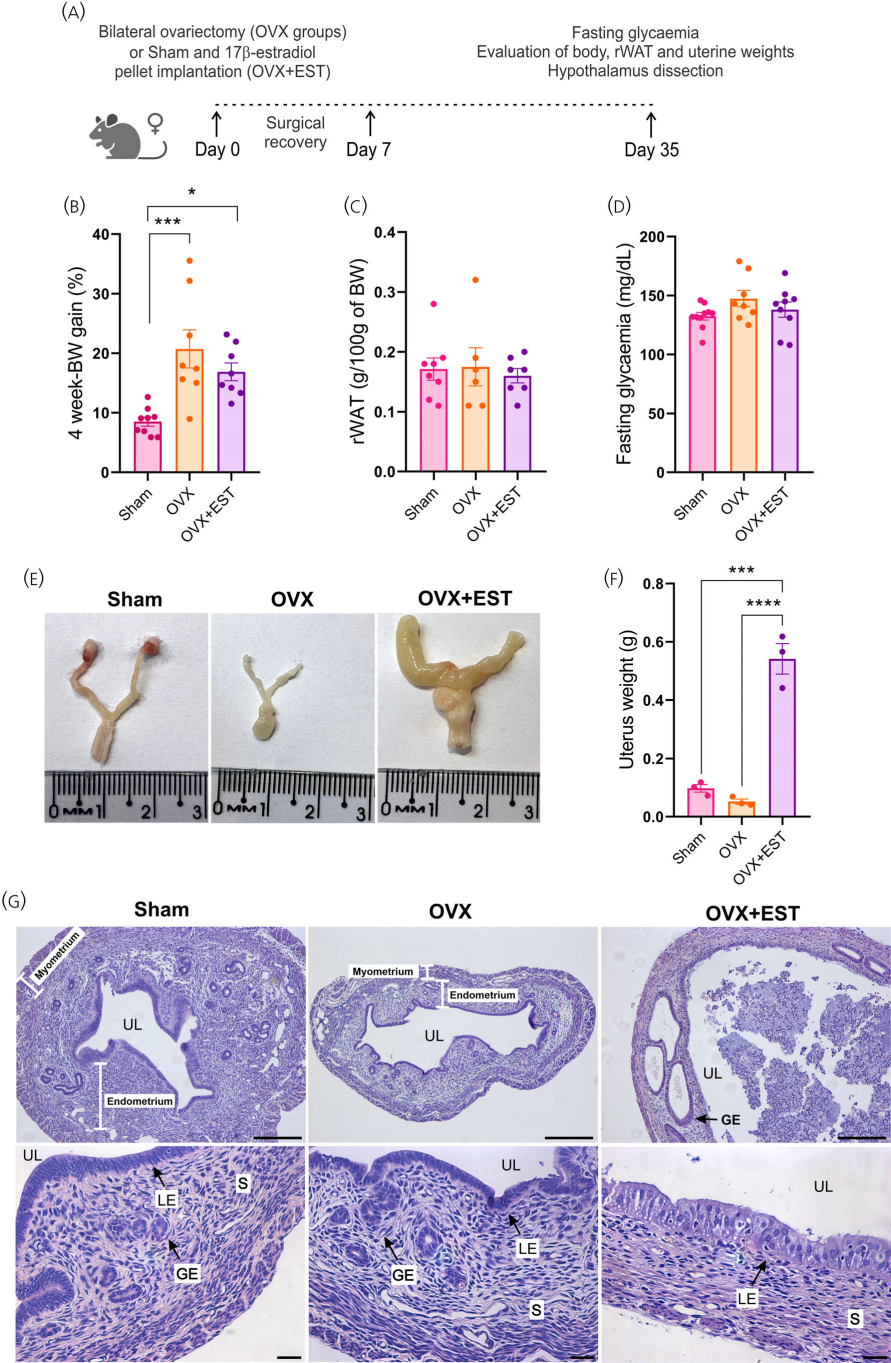
Ovariectomy induces metabolic dysfunction independent of dietary composition. (A) Schematic representation of the experimental design. (B) Percentage of body weight gain during 4 weeks after the surgical removal of the ovaries (OVX) surgical procedure. (C) Weight of white adipose tissue (retroperitoneal depot, retroperitoneal white adipose tissue [rWAT]) at the end of the experiment. (D) Six‐hour fasting blood glucose levels. (E) Representative macroscopic images of the uterus collected at euthanasia. (F) Uterine weight measurements. (G) Representative histological images of uterine horns (hematoxylin and eosin staining). Data are expressed as mean ± standard error of the mean of 5–10 mice per group, except for uterine weight (*N* = 3 per group). Statistical analyses were performed using one‐way ANOVA, followed by Tukey's post hoc test. *p < .05, ***p < .001, *p* < .00001, compared with the Sham group. BW, body weight; EST, estradiol; GE, glandular epithelium; LE, luminal epithelium; S, stroma; UL, uterine lumen.

Next, to isolate the role of ovarian hormones in hypothalamic chemokine expression, females exclusively fed on SD underwent bilateral ovariectomized (OVX) with or without EST replacement (Figure 5A). Both and OVX + EST mice showed increased BW compared with Sham controls (*p* < .05; Figure 5B,C), without significant changes in rWAT depot weight. Fasting glycemia (Figure 5D) and total caloric intake (data not shown) remained unchanged.

Macroscopic analysis showed uterus with visible ovaries in intact females (Sham group), whereas OVX uterus was markedly reduced, thin, and atrophic. EST replacement (OVX + EST) restored uterine growth, resulting in enlarged and hypertrophic organs (Figure 5E). Consistently, uterine weight was lower in OVX compared to Sham, although without statistical significance, while EST replacement (OVX + EST) significantly increased uterine weight relative to both Sham and OVX groups (Figure 5F). Histologically, the uterus from intact females displayed preserved architecture with a well‐developed endometrium, abundant glands, and columnar epithelium. Bilateral ovariectomy resulted in endometrial atrophy with reduced thickness, fewer glands, and lower epithelium. EST treatment reversed these changes, increasing epithelial height and glandular development compared to the OVX group (Figure 5G). Altogether, these morphological, histological, and quantitative findings confirm that the ovariectomy and hormone replacement protocol was effective.

**FIGURE 6:**
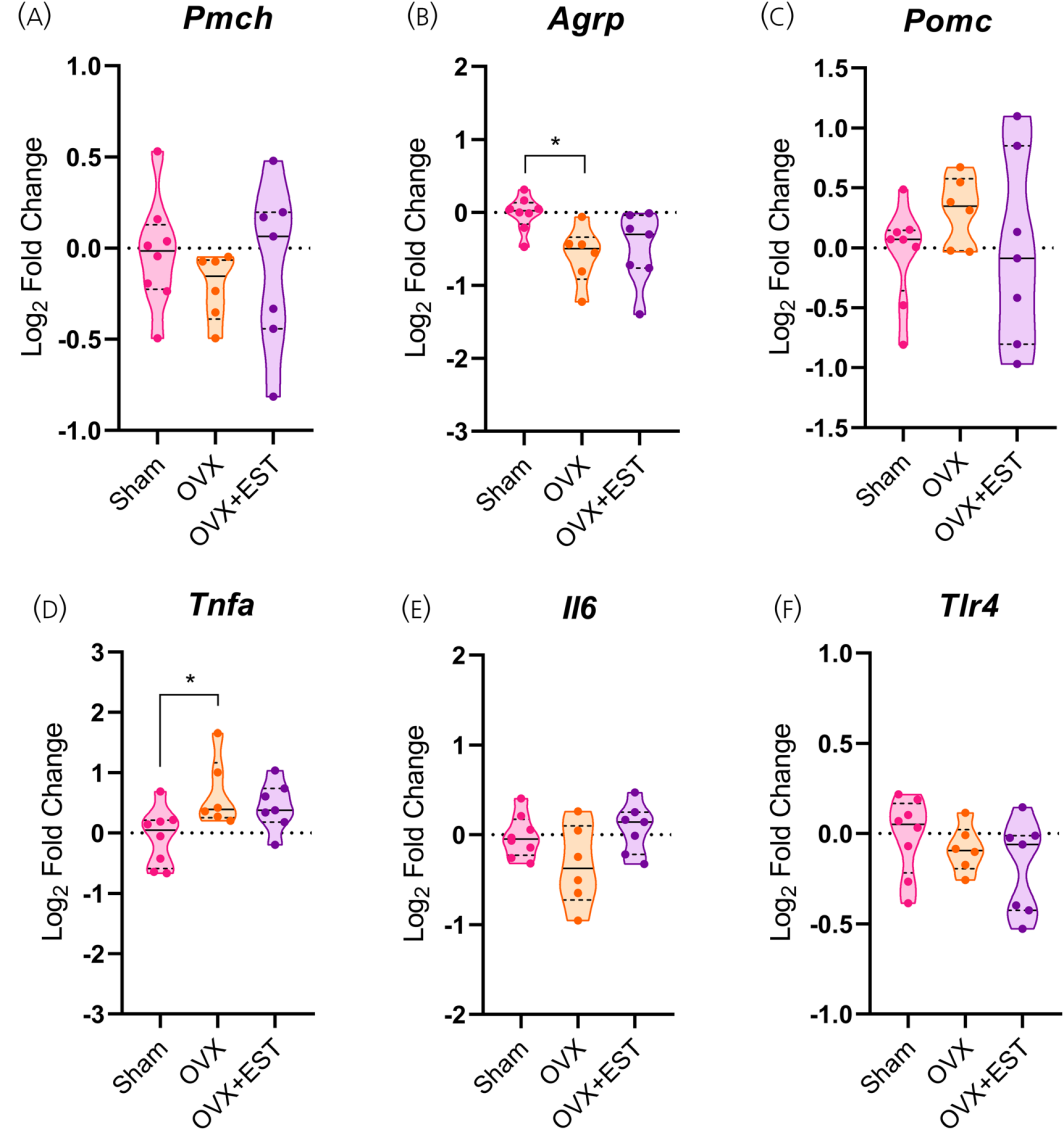
Ovarian hormone deficiency modulates hypothalamic neuropeptides and inflammatory markers. (A–C) Hypothalamic mRNA levels of neuropeptides. (D–F) Hypothalamic mRNA levels of inflammatory markers. Data are shown as violin plots with median and quartiles for five to seven mice per group. qPCR data (log_2_ fold change) were analyzed using one‐way ANOVA, followed by Tukey's post hoc tests. *p < .05 compared with the Sham group. EST, estradiol; OVX, ovariectomized.

The absence of ovarian hormones induced alterations in the hypothalamic transcript levels of the orexigenic neuropeptide *Agrp*, which was reduced in OVX females (*p* < .05; Figure 6B), whereas *Pmch* and *Pom*c expression remained unchanged (Figure 6A–C). These changes were accompanied by signs of inflammatory activation, as indicated by increased *Tnfa* expression in OVX females (*p* < .05; Figure 6D). No differences were detected in *Il6* or *Tlr4* expression levels (Figure 6E,F).

### 3.4 Hypothalamic chemokine profile is influenced by ovarian hormones

**FIGURE 7:**
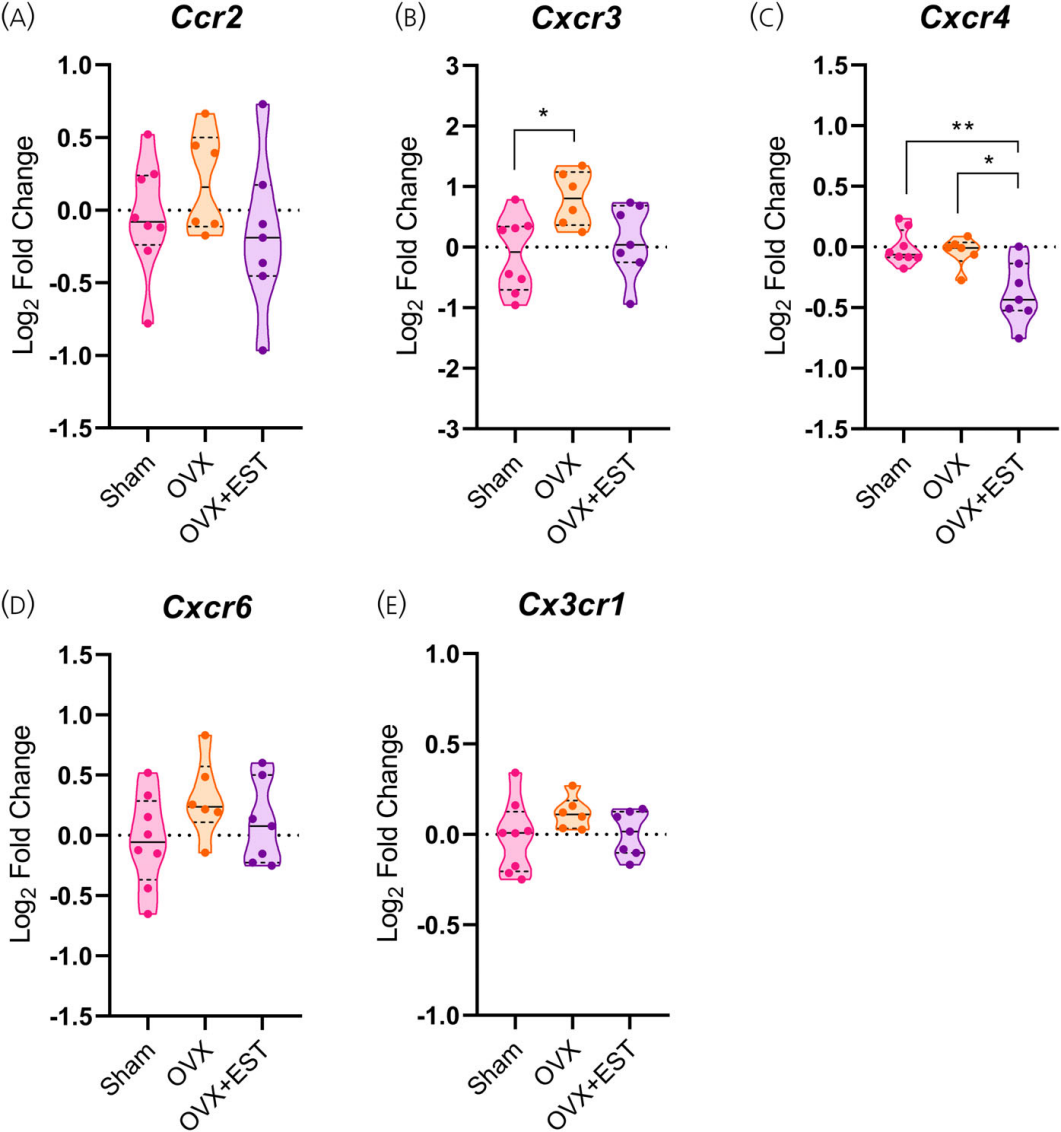
Cxcr3 expression in the hypothalamus is modulated by ovarian hormones. (A–E) Hypothalamic mRNA levels of chemokine receptors. Data are shown as violin plots with median and quartiles for five to seven mice per group. qPCR data (log_2_ fold change) were analyzed using one‐way ANOVA, followed by Tukey's post hoc tests. *p < .05, **p < .01 compared with the indicated groups. EST, estradiol; OVX, ovariectomized.

**FIGURE 8:**
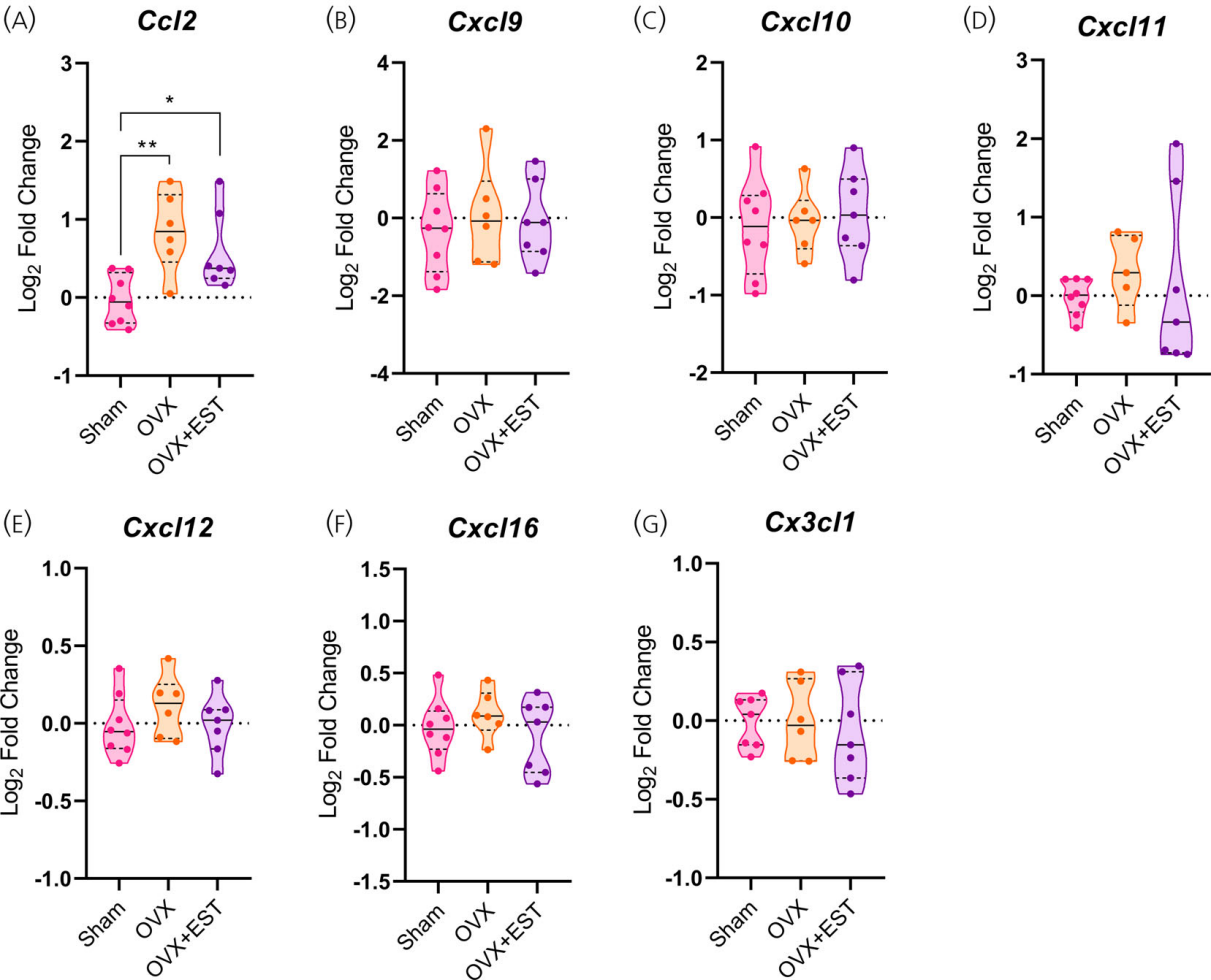
Ovariectomy results in increased *Cxcr3* expression in the hypothalamus. (A–G) Hypothalamic mRNA levels of chemokine receptors. Data are shown as violin plots with median and quartiles for five to seven mice per group. qPCR data (log_2_ fold change) were analyzed using one‐way ANOVA, followed by Tukey's post hoc tests. *p < .05, **p < .01 compared with the Sham group. EST, estradiol; OVX, ovariectomized.

Analysis of hypothalamic chemokine expression in OVX females and their controls revealed a significant increase in *Ccl2* mRNA levels in both OVX and OVX + EST groups (*p* < .001 and *p* < .05, respectively; Figure 8A), whereas *Ccr2* expression remained unchanged (Figure 7A). Consistent with findings from females at 14 and 28 days of HFD, *Cxcr3* expression was upregulated in the OVX group (*p* < .05; Figure 7B), with no differences detected in *Cxcl9*, *Cxcl10*, or *Cxcl11* (Figure 8B–D). Interestingly, *Cxcr4* expression increased in OVX + EST females compared with both Sham and OVX groups (*p* < .05–.01; Figure 7C), while other chemokine receptors and their respective ligands remained unaltered (Figures 7D,E and 8D,E). Overall, these results indicate that the absence of ovarian hormones alters hypothalamic chemokine and inflammatory profiles in females, fostering a pro‐inflammatory state and metabolic dysregulation that are only modestly mitigated by EST replacement.

## 4 DISCUSSION

This study is the first to characterize how short‐ and long‐term exposure to a HFD modulates the hypothalamic chemokine profile in male and female mice, highlighting the role of ovarian hormones in this process. In line with previous studies showing greater male vulnerability to diet‐induced metabolic dysfunction,^20^, ^21^, ^22^, ^23^ we observed that males gained more BW and adipose mass than females under the same dietary conditions. Several studies have reported that HFD exposure rapidly disrupts the homeostatic balance of hypothalamic anorexigenic and orexigenic neurons.^4^, ^24^ In our study, we observed a sex‐specific effect primarily in *Pmch*, which encodes melanin‐concentrating hormone (MCH), reinforcing the influence of sex on MCH regulation, as recently highlighted by Kuebler et al.^25^ Regarding other neuropeptides, our data align with previous reports demonstrating a transient, time‐dependent compensatory response to HFD in both males and females.^20^, ^22^

Our results in OVX females further support the critical role of ovarian hormones in metabolic regulation. Consistent with previous studies,^26^, ^27^, ^28^, ^29^ OVX females exhibited increases in BW accompanied by modifications in hypothalamic neuropeptide expression,^30^, ^31^ despite no differences in total caloric intake compared with Sham controls. In OVX females, we observed a reduction in *Agrp* expression, with no significant changes in *Pomc* levels. In our previous study, analyses of HFD‐fed OVX females revealed lower hypothalamic *Agrp* and higher *Pomc* expression.^17^ These data suggest that *Pomc* upregulation in females occurs primarily through the interaction between ovarian hormone deficiency and HFD, whereas *Agrp* regulation appears to depend on both diet and ovarian hormones through distinct mechanisms. These metabolic data indicate that the absence of ovarian hormones renders females susceptible to metabolic alterations and dysregulated neuropeptide expression, similar to changes observed in chronically HFD‐fed mice, highlighting their pivotal role in protecting hypothalamic function and maintaining energy homeostasis.

We also investigated how biological sex and the duration of HFD exposure modulate the inflammatory response in the hypothalamus. After just 1 day of HFD feeding, male mice exhibited higher hypothalamic *Tnfa* expression compared with females, suggesting an earlier activation of glial cells, as this cytokine is primarily released by microglia in response to excessive saturated fatty acid intake.^7^ This finding is consistent with Cansell et al.^24^ who reported rapid glial activation and increased inflammatory cytokine release in males within only a few hours of HFD exposure. In females, hypothalamic Tnfα expression was increased in OVX mice and restored by estrogen replacement, whereas HFD‐fed intact females showed no changes in this pro‐inflammatory cytokine regardless of HFD duration. Interestingly, in our previous study using OVX mice fed an HFD,^17^ no differences were observed between OVX and Sham groups, suggesting that Tnfα modulation in females is primarily hormone‐dependent rather than directly driven by dietary fat exposure. This apparent discrepancy, together with our previous observations in OVX mice under HFD conditions, suggests that the effects of ovarian hormone deficiency on hypothalamic inflammation are context‐dependent and may be attenuated under chronic metabolic challenge.

Beyond *Tnfa*, we identified additional inflammatory markers showing sex‐specific regulation. Toll‐like receptor 4 (TLR4), a key sensor of dietary saturated fats in the hypothalamus, plays a central role in triggering microglial activation and inflammatory signaling.^32^, ^33^, ^34^ In the present study, HFD‐fed females displayed reduced Tlr4 expression during the early phase of HFD exposure, suggesting a rapid, adaptive protection against inflammatory stimuli. However, *Tlr4* levels did not differ between Sham and OVX females, indicating that ovarian hormones modulate hypothalamic inflammation through mechanisms beyond TLR4 signaling. Interestingly, Curtis et al.^35^ reported that OVX‐induced changes in *Tlr4* expression are nucleus‐specific, with reductions in the ARC but no alterations in the paraventricular nucleus. Since we measured transcript levels in the whole hypothalamus, this broader approach may have masked such nucleus‐specific effects, representing a limitation of the present study.

Regarding the hypothalamic chemokine profile, HFD‐fed females exhibited a progressive reduction in *Cx3cl1* and its receptor *Cx3cr1* from Day 3 to Day 28, suggesting a sustained disruption of neuron–microglia communication. Interestingly, this pattern contrasts with findings from studies using longer HFD exposure (18 weeks), in which females displayed increased hypothalamic CX3CL1/CX3CR1 signaling, a response proposed to counteract metabolic impairments in this sex.^36^ In our previous work, we sorted hypothalamic CX3CR1^+^ cells by FACS and found no major sex differences in cell number after 4 weeks of HFD.^17^ Thus, the HFD‐induced changes in both *Cx3cl1* and *Cx3cr1* mRNA observed here likely reflect impaired neuron–microglia communication, potentially contributing to attenuated microglial pro‐inflammatory cytokine release in females.

The anatomic position of the hypothalamus, adjacent to the third ventricle and near the fenestrated capillaries of the ME, facilitates chemokine‐mediated immune cell infiltration under HFD conditions.^37^ In experimental models of diet‐induced obesity, CCL2 (also known as MCP‐1) is a key chemokine in this process, showing raised expression in various tissues.^38^, ^39^ In the present study, we observed that hypothalamic CCL2/CCR2 signaling exhibited a dynamic, biphasic response to HFD exposure. Initially, both *Ccl2* and *Ccr2* expression were downregulated during the acute phase (Days 1–3), followed by recovery or upregulation during chronic feeding (Days 14–28). This transient pattern likely represents an adaptive mechanism; the early suppression may restrain excessive neuroinflammation, while subsequent reactivation facilitates CCR2^+^ monocyte recruitment that contributes to the chronic inflammatory state. Supporting this interpretation, previous studies have shown that CCR2^+^ cell infiltration into the hypothalamus increases progressively with HFD duration, with substantial parenchymal accumulation evident by 4 weeks.^15^, ^17^, ^18^

Lainez et al.^18^ quantified cluster of differentiation 11b and high levels of cluster of differentiation 45 (CD11b^+^CD45^high^) macrophages in the hypothalamus of 12‐week HFD‐fed mice and found greater accumulation in males than females, correlating with worse metabolic dysfunction in males. Regarding CCR2^+^ cells, we previously found similar frequencies in the hypothalamic parenchyma of males and females after 4 weeks of HFD using flow cytometry.^17^ Consistent with these cellular data, we detected no sex differences in *Ccr2* or *Ccl2* mRNA expression during either acute or chronic HFD exposure. Notably, despite similar CCR2/CCL2 expression between sexes, our previous transcriptomic analysis revealed sex‐specific gene expression profiles within the CCR2^+^ infiltrating cells themselves, particularly in chemotaxis and interferon‐γ pathways.^17^ These findings suggest that functional differences between male and female immune responses extend beyond the transcriptional regulation of chemokine ligands and receptors, potentially involving distinct cellular activation states or effector functions.

An important feature of the infiltrating CCR2^+^ cell population in the central nervous system (CNS) is a subset that co‐expresses CXCR3, a chemokine receptor found on monocytes and activated Tregs. We have previously shown that infiltration of CCR2^+^CXCR3^hi^ cells into the hypothalamus attenuates HFD‐induced metabolic impairments,^17^ suggesting a protective role for this specific immune cell subset. Here, to investigate sex‐specific regulation of CXCR3 during HFD exposure, we have also analyzed its hypothalamic transcript levels. While most chemokines showed similar patterns between sexes, *Cxcr3* was a notable exception; females consistently exhibited higher expression, particularly during later stages of HFD. Interestingly, OVX females also displayed elevated hypothalamic *Cxcr3*, which was restored by estrogen replacement. The consistent upregulation of CXCR3 in both HFD‐fed and OVX females suggests a controlled recruitment of a subset of immune cells that may aid resolution rather than exacerbate inflammation. This pattern aligns with the milder inflammatory phenotype in females and indicates hormone‐dependent modulation of chemotactic signaling that supports metabolic protection.

In conclusion, our findings show that HFD induces dynamic, sex‐specific changes in hypothalamic chemokine signaling. While the CCL2–CCR2 axis was strongly modulated by HFD in both sexes, females exhibited lower *Tnfa* levels at HFD onset, a progressive reduction in *Cx3cl1* and *Cx3cr1* transcripts, and increased *Cxcr3* expression during chronic stages, likely contributing to their early metabolic resilience. Ovarian hormone deficiency abolished this protective profile, shifting females toward a pro‐inflammatory state resembling males. These results highlight ovarian hormones as critical regulators of hypothalamic chemokine networks and metabolic homeostasis, underscoring the importance of sex‐dependent mechanisms in neuroimmune regulation.

Importantly, our quantitative PCR‐based approach captures broad transcriptional dynamics, but future studies using nucleus‐specific analyses and cellular localization will be crucial to resolve region‐specific mechanisms in HFD and OVX models. Additionally, estrous cycle stage was not monitored in the HFD time‐course or Sham cohorts, which may contribute to within‐group variability in females and represents a limitation to be addressed in future studies. It should also be noted that the estrogen dose employed in the OVX replacement model may have exceeded physiological levels, as indicated by the marked increase in uterine mass observed relative to the Sham group, which represents an additional limitation of the present study. Targeted hormone replacement studies could further clarify the roles of estrogen, progesterone, and other ovarian factors in shaping these sex‐specific neuroimmune and metabolic adaptations.

## AUTHOR CONTRIBUTIONS

**NFM**: designed and planned the study, wrote the manuscript, and prepared the figures; **AGBA** and **GAdS**: performed most of the experiments; **IVSC**, **VSM**, **LSB**, **NPM**, and **EdAS**: contributed to tissue harvesting and/or surgical procedures; **LAV**: supervised **EdAS** and **LBS** during their doctoral programs and contributed to data discussion. All authors read and approved the final manuscript.

## FUNDING INFORMATION

This research was funded by Sao Paulo Research Foundation (FAPESP) (2013/07607‐8 and 2022/06282‐7). Alexia Guimaraes Batista Augusto and Giovanna Ariozi dos Santos received fellowships from FAPESP (2023/11393‐5 and 2024/18774‐7, respectively). Open access publishing facilitation was provided by Brazilian Federal Agency for Support and Evaluation of Graduate Education (CAPES), through its transformative “Read and Publish” agreement with Wiley.

## CONFLICTS OF INTEREST

The authors declare no conflict of interest.

## Supporting information


**Table S1.** List of analyzed genes and their respective assay IDs.

## Data Availability

Data will be available upon request to the corresponding author, Natalia Ferreira Mendes (natocrc@unicamp.br).

## References

[1] Schwartz MW , Woods SC , Porte D Jr , Seeley RJ , Baskin DG . Central nervous system control of food intake. Nature. 2000;404(6778):661‐671. doi:10.1038/35007534 10766253

[2] Myers MG Jr , Affinati AH , Richardson N , Schwartz MW . Central nervous system regulation of organismal energy and glucose homeostasis. Nat Metab. 2021;3(6):737‐750. doi:10.1038/s42255-021-00408-5 (Erratum in Nat Metab: 2021 Jul;3(7):1033). 34188225

[3] De Souza CT , Araujo EP , Bordin S , et al. Consumption of a fat‐rich diet activates a proinflammatory response and induces insulin resistance in the hypothalamus. Endocrinology. 2005;146(10):4192‐4199. doi:10.1210/en.2004-1520 16002529

[4] Thaler JP , Yi CX , Schur EA , et al. Obesity is associated with hypothalamic injury in rodents and humans. J Clin Invest. 2012;122(1):153‐162. doi:10.1172/JCI59660 (Erratum in: J Clin Invest 2012 Feb 1;122(2):778). 22201683 PMC3248304

[5] Zagmutt S , Rodriguez‐Garcia M , Bolaños‐Hurtado M , Reguera AC , Casals N , Rodriguez‐Rodriguez R . Redefining the timeline: a three‐phase framework of hypothalamic microinflammation in metabolic disease. Rev Endocr Metab Disord. 2025;27:435‐456. doi:10.1007/s11154-025-09992-3 41065953 PMC13246836

[6] Engel DF , Velloso LA . The timeline of neuronal and glial alterations in experimental obesity. Neuropharmacology. 2022;208:108983. doi:10.1016/j.neuropharm.2022.108983 35143850

[7] Valdearcos M , Robblee MM , Benjamin DI , Nomura DK , Xu AW , Koliwad SK . Microglia dictate the impact of saturated fat consumption on hypothalamic inflammation and neuronal function. Cell Rep. 2014;9(6):2124‐2138. doi:10.1016/j.celrep.2014.11.018 25497089 PMC4617309

[8] Baufeld C , Osterloh A , Prokop S , Miller KR , Heppner FL . High‐fat diet‐induced brain region‐specific phenotypic spectrum of CNS resident microglia. Acta Neuropathol. 2016;132(3):361‐375. doi:10.1007/s00401-016-1595-4 27393312 PMC4992033

[9] Lee CH , Kim HJ , Lee YS , et al. Hypothalamic macrophage inducible nitric oxide synthase mediates obesity‐associated hypothalamic inflammation. Cell Rep. 2018;25(4):934‐946.e5. doi:10.1016/j.celrep.2018.09.070 30355499 PMC6284237

[10] Jais A , Solas M , Backes H , et al. Myeloid‐cell‐derived VEGF maintains brain glucose uptake and limits cognitive impairment in obesity. Cell. 2016;165(4):882‐895. doi:10.1016/j.cell.2016.03.033 (Erratum in: Cell 2016 Aug 25;166(5):1338–1340). 27565353

[11] Mendes NF , Velloso LA . Perivascular macrophages in high‐fat diet‐induced hypothalamic inflammation. J Neuroinflammation. 2022;19(1):136. doi:10.1186/s12974-022-02519-6 35681242 PMC9185933

[12] Münzberg H , Flier JS , Bjørbaek C . Region‐specific leptin resistance within the hypothalamus of diet‐induced obese mice. Endocrinology. 2004;145(11):4880‐4889. doi:10.1210/en.2004-0726 15271881

[13] Carmo‐Silva S , Cavadas C . Hypothalamic dysfunction in obesity and metabolic disorders. Adv Neurobiol. 2017;19:73‐116. doi:10.1007/978-3-319-63260-5_4 28933062

[14] Schwartz MW , Porte D Jr . Diabetes, obesity, and the brain. Science. 2005;307(5708):375‐379. doi:10.1126/science.1104344 15662002

[15] Valdearcos M , Douglass JD , Robblee MM , et al. Microglial inflammatory signaling orchestrates the hypothalamic immune response to dietary excess and mediates obesity susceptibility. Cell Metab. 2017;26(1):185‐197.e3. doi:10.1016/j.cmet.2017.05.015 (Erratum in: Cell Metab 2018 Jun 05;27(6):1356). 29874568

[16] Becker M , Kälin S , Neubig AH , et al. Regulatory T cells in the mouse hypothalamus control immune activation and ameliorate metabolic impairments in high‐calorie environments. Nat Commun. 2025;16(1):2744. doi:10.1038/s41467-025-57918-z 40113758 PMC11926360

[17] Mendes N , Zanesco A , Aguiar C , et al. CXCR3‐expressing myeloid cells recruited to the hypothalamus protect against diet‐induced body mass gain and metabolic dysfunction. Elife. 2024;13:RP95044. doi:10.7554/eLife.95044 39535032 PMC11560133

[18] Lainez NM , Jonak CR , Nair MG , et al. Diet‐induced obesity elicits macrophage infiltration and reduction in spine density in the hypothalami of male but not female mice. Front Immunol. 2018;9:1992. doi:10.3389/fimmu.2018.01992 30254630 PMC6141693

[19] Claret M , Haddad‐Tóvolli R . Sexual dimorphism in the development and function of the melanocortin system. Rev Endocr Metab Disord. 2025;27:561‐572. doi:10.1007/s11154-025-09983-4 40555875

[20] Dreux V , Lefebvre C , Breemeersch CE , et al. Sex‐dependent effects of a high‐fat diet on the hypothalamic response in mice. Biol Sex Differ. 2025;16(1):17. doi:10.1186/s13293-025-00699-3 40001261 PMC11854408

[21] Casimiro I , Stull ND , Tersey SA , Mirmira RG . Phenotypic sexual dimorphism in response to dietary fat manipulation in C57BL/6J mice. J Diabetes Complications. 2021;35(2):107795. doi:10.1016/j.jdiacomp.2020.107795 33308894 PMC7856196

[22] Oraha J , Enriquez RF , Herzog H , Lee NJ . Sex‐specific changes in metabolism during the transition from chow to high‐fat diet feeding are abolished in response to dieting in C57BL/6J mice. Int J Obes (Lond). 2022;46(10):1749‐1758. doi:10.1038/s41366-022-01174-4 35794191 PMC9492540

[23] Ribas V , Morón‐Ros S , Marí H , et al. Diet‐induced obesity disrupts sexually dimorphic gene expression in mice. Am J Physiol Cell Physiol. 2025;329(4):C987‐C1003. doi:10.1152/ajpcell.00098.2025 40758559

[24] Cansell C , Stobbe K , Sanchez C , et al. Dietary fat exacerbates postprandial hypothalamic inflammation involving glial fibrillary acidic protein‐positive cells and microglia in male mice. Glia. 2021;69(1):42‐60. doi:10.1002/glia.23882 32659044

[25] Kuebler IRK , Suárez M , Wakabayashi KT . Sex differences and sex‐specific regulation of motivated behavior by melanin‐concentrating hormone: a short review. Biol Sex Differ. 2024;15(1):33. doi:10.1186/s13293-024-00608-0 38570844 PMC10993549

[26] da Silva RP , Zampieri TT , Pedroso JA , et al. Leptin resistance is not the primary cause of weight gain associated with reduced sex hormone levels in female mice. Endocrinology. 2014;155:4226‐4236. 25144922 10.1210/en.2014-1276

[27] Meneyrol K , Estévez‐Salguero Á , González‐García I , et al. Ovarian insufficiency impairs glucose‐stimulated insulin secretion through activation of hypothalamic de novo ceramide synthesis. Metabolism. 2021;123:154846. doi:10.1016/j.metabol.2021.154846 34371064

[28] Rogers NH , Perfield JW 2nd , Strissel KJ , Obin MS , Greenberg AS . Reduced energy expenditure and increased inflammation are early events in the development of ovariectomy‐induced obesity. Endocrinology. 2009;150(5):2161‐2168. doi:10.1210/en.2008-1405 19179442 PMC2671894

[29] Zengin A , Nguyen AD , Wong IP , et al. Neuropeptide Y mediates the short‐term hypometabolic effect of estrogen deficiency in mice. Int J Obes (Lond). 2013;37(3):390‐398. doi:10.1038/ijo.2012.71 22565420

[30] Wang W , Yang Q , Zhou C , et al. Transcriptomic changes in the hypothalamus of ovariectomized mice: data from RNA‐seq analysis. Ann Anat. 2022;241:151886. doi:10.1016/j.aanat.2022.151886 35032566

[31] Ainslie DA , Morris MJ , Wittert G , Turnbull H , Proietto J , Thorburn AW . Estrogen deficiency causes central leptin insensitivity and increased hypothalamic neuropeptide Y. Int J Obes Relat Metab Disord. 2001;25(11):1680‐1688. doi:10.1038/sj.ijo.0801806 11753591

[32] Milanski M , Arruda AP , Coope A , et al. Inhibition of hypothalamic inflammation reverses diet‐induced insulin resistance in the liver. Diabetes. 2012;61(6):1455‐1462. doi:10.2337/db11-0390 22522614 PMC3357298

[33] Masson GS , Nair AR , Dange RB , Silva‐Soares PP , Michelini LC , Francis J . Toll‐like receptor 4 promotes autonomic dysfunction, inflammation and microglia activation in the hypothalamic paraventricular nucleus: role of endoplasmic reticulum stress. PLoS One. 2015;10(3):e0122850. doi:10.1371/journal.pone.0122850 25811788 PMC4374971

[34] Prévost M , Crépin D , Rifai SA , et al. The resistin/TLR4/miR‐155‐5p axis: a novel signaling pathway in the onset of hypothalamic neuroinflammation. J Neuroinflammation. 2025;22(1):198. doi:10.1186/s12974-025-03522-3 40759954 PMC12323067

[35] Curtis KS , McCracken K , Espinosa E , Ong J , Buck DJ , Davis RL . Temporal and site‐specific changes in central neuroimmune factors during rapid weight gain after ovariectomy in rats. Neurochem Res. 2018;43(9):1802‐1813. doi:10.1007/s11064-018-2596-6 30030770

[36] Dorfman MD , Krull JE , Douglass JD , et al. Sex differences in microglial CX3CR1 signalling determine obesity susceptibility in mice. Nat Commun. 2017;8:14556. doi:10.1038/ncomms14556 28223698 PMC5322503

[37] Rodríguez EM , Blázquez JL , Guerra M . The design of barriers in the hypothalamus allows the median eminence and the arcuate nucleus to enjoy private milieus: the former opens to the portal blood and the latter to the cerebrospinal fluid. Peptides. 2010;31(4):757‐776. doi:10.1016/j.peptides.2010.01.003 20093161

[38] Carlin JL , Grissom N , Ying Z , Gomez‐Pinilla F , Reyes TM . Voluntary exercise blocks Western diet‐induced gene expression of the chemokines CXCL10 and CCL2 in the prefrontal cortex. Brain Behav Immun. 2016;58:82‐90. doi:10.1016/j.bbi.2016.07.161 27492632 PMC5352157

[39] Chen KE , Lainez NM , Coss D . Sex differences in macrophage responses to obesity‐mediated changes determine migratory and inflammatory traits. J Immunol. 2021;206(1):141‐153. doi:10.4049/jimmunol.2000490 33268480 PMC8903060

