# Supplementary material for: Ovarian hormones and high‐fat diet duration distinctively modulate hypothalamic chemokine profile
Source: J Neuroendocrinol. 2026 Jun 25;38(7):e70215. doi: 10.1111/jne.70215 (PMC13296257; doi:10.1111/jne.70215)
Supplement: Supplementary file 1 — Table S1. List of analyzed genes and their respective assay IDs. [file JNE-38-e70215-s001.docx]

**Supplementary Table S1.** List of analyzed genes and their respective Assay IDs.

| **Gene** | **Assay ID** |
| --- | --- |
| *Cxcr3* | Mm99999054_s1 |
| *Cxcl9* | Mm00434946_m1 |
| *Cxcl10* | Mm00445235_m1 |
| *Cxcl11* | Mm00444662_m1 |
| *Ccl2* | Mm00441242_m1 |
| *Cxcr4* | Mm01996749_s1 |
| *Cxcl12* | Mm00445553_m1 |
| *Cxcr6* | Mm02620517_s1 |
| *Cxcl16* | Mm00469712_m1 |
| *Cx3cl1* | Mm00436454_m1 |
| *Pomc* | Mm00435874_m1 |
| *Agrp* | Mm00475829_g1 |
| *Pmch* | Mm01242886_g1 |
| *Tnfa* | Mm00443258_m1 |
| *Il6* | Mm00446190_m1 |
| *Tlr4* | Mm00445273_m1 |
